# Study of Model Uncertainties Influence on the Impact Point Dispersion for a Gasodynamicaly Controlled Projectile

**DOI:** 10.3390/s22093257

**Published:** 2022-04-24

**Authors:** Mariusz Jacewicz, Piotr Lichota, Dariusz Miedziński, Robert Głębocki

**Affiliations:** Division of Mechanics, Institute of Aeronautics and Applied Mechanics, Warsaw University of Technology, Nowowiejska 24, 00-665 Warsaw, Poland; mariusz.jacewicz@pw.edu.pl (M.J.); piotr.lichota@pw.edu.pl (P.L.); robert.glebocki@pw.edu.pl (R.G.)

**Keywords:** flight simulation, dispersion analysis, rocket, pulse jet control

## Abstract

The article presents the analysis of the impact point dispersion reduction using lateral correction thrusters. Two types of control algorithms are used and four sources of uncertainties are taken into account: aerodynamic parameters, thrust curve, initial conditions and IMU errors. The Monte Carlo approach was used for simulations and Circular Error Probable was used as a measure of dispersion. Generic rocket mathematical and simulation model was created in MATLAB/Simulink 2020b environment. Results show that the use of control algorithms greatly reduces the impact point dispersion.

## 1. Introduction

Determining and lowering the impact point dispersion of an artillery projectile is an important factor when determining its usefulness and effectiveness. Due to the imperfections of modelling of the flight of such objects, unknowns, simplifications, and uncertainties in model parameters, it is important to take into account the various possibilities of scenarios and determine and quantify the dispersion of possible landing points. Key factors influencing the flight of the object, and the hardest to obtain accurately for modelling, are the aerodynamic characteristics, thrust curve, and initial conditions. To maximize the accuracy of the projectile, various types of control methods, algorithms, and target detection methods are utilized, such as H${}_{\infty }$ guidance law [1], proportional navigation guidance and its modifications [2,3], various optimal control methods [4,5,6,7] or model predictive approach [8].

In [9,10] the impact point dispersion of a lateral pulse jet controlled rocket following a reference trajectory and its robustness to the effects of the measurement noise were studied. Launch conditions’ uncertainties were studied in [11,12]. In [13,14] the impact point dispersion due to manufacturing errors using Monte Carlo method was studied. In [15] the effects of launch condition variability, atmospheric factors, and IMU errors on the guidance accuracy were investigated. The influence of missile initial conditions’ uncertainties and IMU errors on the impact point dispersion using lateral thrusters for control were investigated in [16] and the influence of uncertainties in rocket parameters on the performance of a cold launch were analyzed in [17]. IMU errors and noise impact on the guidance were also investigated in [18,19]. The analysis of the robustness of the control algorithm with respect to uncertainty regarding the launch environment and rocket conditions was presented in [20,21]. Monte Carlo analysis of the impact point dispersion due to the missile parameters and atmospheric conditions’ uncertainties was performed in [22] and the impact point dispersion reduction due to high spin motion was analyzed in [23].

In this paper, the analysis of the impact point dispersion caused by model uncertainties is presented. The uncertainties in the aerodynamic parameters, thrust curve, and initial conditions, as well as the uncertainties caused by the Inertial Measurement Unit model and its errors are analyzed. Two types of control algorithms were tested, Multi-Condition Control Algorithm (MCCA) and modified Proportional Navigation Guidance (mPNG). The analysis was performed using the Monte Carlo approach.

The prepared article is organised as follows: in Section 2 the mathematical model of the rocket is presented in the Section 2.1 with used assumptions and coordinate systems. The dynamic equations of motion, followed by additional kinematic equations are shown in that section. The external loads comprised of aerodynamics, gravity, propulsion, and correction thrusters are presented, and this is followed by the description of inertial parameters, atmosphere, and Inertial Measurement Unit modelling approach. At last, the used control algorithms are shown. In Section 2.2 the simulational model created in MATLAB/Simulink R2020b is presented. Section 3 presents the simulational study and its results, followed by the discussion and interpretation. The paper finishes with a short summary of the conclusions shown in Section 4.

The novelty in this paper is the comparison of the IMU errors’ influence on the accuracy of the two control algorithms utilizing lateral pulse jet control.

The developed numerical simulation might be used in design process of new sensors intended for projectile navigation. This tool might be used for fast prototyping of control schemes that reduces the overall system design process and costs.

## 2. Materials and Methods

### 2.1. Mathematical Model

#### 2.1.1. Assumptions

For creating a mathematical and simulational model of a generic rocket, several assumptions were made. The rocket is modelled as a rigid body with six degrees of freedom and variable inertial parameters. It is controlled by a set of solid rocket motor thrusters, which use does not change the inertial and aerodynamic parameters of the rocket. Atmosphere model is taken from the International Standard Atmosphere [24]. Earth rotation and eccentricity are not modelled, the gravitational acceleration is constant and consistent with WGS-84 model [25]. The mathematical model includes the Inertial Measurement Unit (IMU) comprised of accelerometers and gyroscopes triades, modelled as second-order dynamical systems, with their disturbances that include noise, bias, scale factor and cross coupling as well as g-dependent factor for gyroscopes.

#### 2.1.2. Coordinate Systems

**Figure 1:**
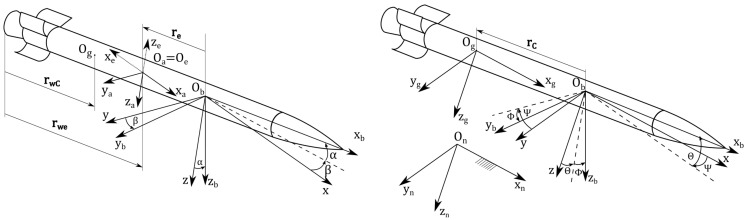
Definition of measuring and aerodynamic coordinate systems, and navigational, gravitational and body coordinate systems.

The coordinate systems used are presented in Figure 1:

- The navigational coordinate system $O_{n}x_{n}y_{n}z_{n}$ is a right-handed, Cartesian coordinate system fixed to earth. Its origin is located at any point and the $O_{n}x_{n}y_{n}$ plane is tangent to the surface of the earth. The $O_{n}x_{n}$ axis points in the direction of a launch rail, $O_{n}z_{n}$ axis in the direction of the gravitational acceleration and $O_{n}y_{n}$ axis completes the right-handed coordinate system.
- The gravitational coordinate system $O_{g}x_{g}y_{g}z_{g}$ is a right-handed, Cartesian moving coordinate system fixed with the rocket. Its origin is located at the center of mass of the rocket and the whole system remains parallel to the navigational system during the whole flight of the rocket.
- The body coordinate system $O_{b}x_{b}y_{b}z_{b}$ is a right-handed Cartesian coordinate system. Its origin is located at any point of the rocket. The $O_{b}x_{b}$ axis is parallel to the rockets longitudinal axis and points forward, $O_{b}y_{b}$ axis points at the right wing and $O_{b}z_{b}$ axis completes the right-handed coordinate system. Orientation of the body coordinate system with respect to the gravitational coordinate system is described by the Euler angles of yaw Ψ, pitch Θ and roll Φ.
- The measuring coordinate system $O_{e}x_{e}y_{e}z_{e}$ is a right-handed, Cartesian coordinate system fixed with the airflow. Its origin is located at any point of the $O_{b}y_{b}z_{b}$ symmetry plane and the position of that point with respect to the $O_{b}x_{b}y_{b}z_{b}$ coordinate system is defined by the vector $r_{e}$. The $O_{e}x_{e}$ axis lies in the direction of the airflow, $O_{e}z_{e}$ axis points upwards and the $O_{e}y_{e}$ axis points to the right.
- The aerodynamic coordinate system $O_{a}x_{a}y_{a}z_{a}$ is the right-handed Cartesian coordinate system fixed with the airflow. The $O_{a}x_{a}$ axis points in the opposite direction that the airflow, $O_{a}z_{a}$ axis points downwards and the $O_{a}y_{a}$ completes the right-handed coordinate system.

#### 2.1.3. Dynamic Equations of Motion

For developing the dynamic equations of motion, the linear and angular momentum change theorems for the rigid body were used. In the non-inertial frame $O_{b}x_{b}y_{b}z_{b}$ with the origin not located at the center of mass, they are given as [26,27]:

(1) $$\frac{\tilde{\delta }\Pi }{\tilde{\delta }t}+\Omega \times \Pi =F_{b}$$

(2) $$\frac{\tilde{\delta }K_{0}}{\tilde{\delta }t}+\Omega \times K_{0}+V_{b}\times \Pi =M_{b}$$

where $V_{b}={\left[\begin{array}{ccc} U & V & W \end{array}\right]}^{T}$ is the velocity vector, $\Omega ={\left[\begin{array}{ccc} P & Q & R \end{array}\right]}^{T}$ is the angular velocity vector, $F_{b}={\left[\begin{array}{ccc} X_{b} & Y_{b} & Z_{b} \end{array}\right]}^{T}$ is the vector of external forces acting on the object, $M_{b}={\left[\begin{array}{ccc} L_{b} & M_{b} & N_{b} \end{array}\right]}^{T}$ is the vector of external torques with respect to point $O_{b}$ and $\frac{\tilde{\delta }}{\tilde{\delta }t}$ is the local derivative. Linear and angular momentum for a rigid body are [26]:

(3) $$\Pi =m\left(V_{b}+\Omega \times r_{C}\right)$$

(4) $$K_{0}=I\Omega +r_{C}\times mV_{b}$$

where *m* is the instantaneous mass of the rocket, I is the instantaneous moment of inertia tensor and $r_{C}$ is the center of mass position with respect to $O_{b}$. The dynamic equations of motion can be written as:

(5) $$m{\dot{V}}_{b}+\dot{\Omega }\times S+\Omega \times mV_{b}+\Omega \times \left(\Omega \times S\right)=F_{b}$$

(6) $$I\dot{\Omega }+S\times {\dot{V}}_{b}+\dot{I}\Omega +\Omega \times I\Omega +\Omega \times \left(S\times V_{b}\right)+V_{b}\times \left(\Omega \times S\right)=M_{b}$$

where $S=mr_{C}$ is the first moment of mass. It must be noted that the propulsion terms resulting from expelling of the propellant by the main motor is included on the right side of above-mentioned equations. In the moments equations the jet damping effect was also omitted because for rocket artillery projectiles it is rather small when compared to aerodynamic damping. After changing cross-products to matrix multiplication, using the skew-symmetric matrix notation, ${\left[\right]}_{x}$, this can be written as:

(7) $$\left[\begin{array}{cc} m1 & -{\left[S\right]}_{x}\\ {\left[S\right]}_{x} & I \end{array}\right]\left[\begin{array}{c} {\dot{V}}_{b}\\ \dot{\Omega } \end{array}\right]+\left[\begin{array}{cc} 0 & 0\\ 0 & \dot{I} \end{array}\right]\left[\begin{array}{c} V_{b}\\ \Omega \end{array}\right]+\left[\begin{array}{cc} {\left[\Omega \right]}_{x} & 0\\ {}[V_{b}{]}_{x} & {\left[\Omega \right]}_{x} \end{array}\right]\left[\begin{array}{cc} m1 & -{\left[S\right]}_{x}\\ {\left[S\right]}_{x} & I \end{array}\right]\left[\begin{array}{c} V_{b}\\ \Omega \end{array}\right]=\left[\begin{array}{c} F_{b}\\ M_{b} \end{array}\right]$$

where 0 is the zero matrix and 1 is the unit matrix. In the short form this is:

(8) $$A\dot{x}+\dot{A}x+\omega \mathrm{Ax}=F_{B}$$

where the state vector has the form $x={\left[\begin{array}{cccccc} U & V & W & P & Q & R \end{array}\right]}^{T}$. This equation can be then numerically integrated to obtain the state vector.

#### 2.1.4. Orientation

For determining object orientation, the quaternion algebra was used. Quaternion describes the orientation of the object in terms of rotation around a specific axis and is written as [28,29]:

(9) $$e=e_{0}+e_{1}i+e_{2}j+e_{3}k$$

where $e_{0},e_{1},e_{2},e_{3}$ are the real numbers and i,j,k are the axes versors. The real parts of the quaternion can be written in terms of rotation axes direction cosines $E_{x},E_{y},E_{z}$ and rotation angle $\delta_{E}$ and are presented in Equations (10)–().

(10) $$\begin{array}{ccc} e_{0} & = & \cos\frac{\delta_{E}}{2} \end{array}$$

(11) $$\begin{array}{ccc} e_{1} & = & E_{x}\sin\frac{\delta_{E}}{2} \end{array}$$

(12) $$\begin{array}{ccc} e_{2} & = & E_{y}\sin\frac{\delta_{E}}{2} \end{array}$$

(13) $$\begin{array}{ccc} e_{3} & = & E_{z}\sin\frac{\delta_{E}}{2} \end{array}$$

The kinematic equation for the rate of change of the quaternion is given as [29,30]:

(14) $$\left[\begin{array}{c} {\dot{e}}_{0}\\ {\dot{e}}_{1}\\ {\dot{e}}_{2}\\ {\dot{e}}_{3} \end{array}\right]=-\frac{1}{2}\left[\begin{array}{cccc} 0 & P & Q & R\\ -P & 0 & -R & Q\\ -Q & R & 0 & -P\\ -R & -Q & P & 0 \end{array}\right]\left[\begin{array}{c} e_{0}\\ e_{1}\\ e_{2}\\ e_{3} \end{array}\right]-kE\left[\begin{array}{c} e_{0}\\ e_{1}\\ e_{2}\\ e_{3} \end{array}\right]$$

where *k* is the feedback coefficient and *E* is the bounding equation violation coefficient $E={\left|e\right|}^{2}-1$. It was assumed that k=1. Quaternions can be used to calculate the transformation matrix from the body to the navigation coordinate system as [29]:

(15) $$\Lambda =\left[\begin{array}{ccc} e_{0}^{2}+e_{1}^{2}-e_{2}^{2}-e_{3}^{2} & 2(e_{1}e_{2}-e_{0}e_{3}) & 2(e_{0}e_{2}+e_{1}e_{3})\\ 2(e_{0}e_{3}+e_{1}e_{2}) & e_{0}^{2}-e_{1}^{2}+e_{2}^{2}-e_{3}^{2} & 2(e_{2}e_{3}-e_{0}e_{1})\\ 2(e_{1}e_{3}-e_{0}e_{2}) & 2(e_{0}e_{1}+e_{2}e_{3}) & e_{0}^{2}-e_{1}^{2}-e_{2}^{2}+e_{3}^{2} \end{array}\right]$$

and orientation angles of roll, pitch, and yaw, given as [29,30]:

(16) $$\begin{array}{ccc} \Phi & = & \arctan\frac{2(e_{0}e_{1}+e_{2}e_{3})}{e_{0}^{2}-e_{1}^{2}-e_{2}^{2}+e_{3}^{2}} \end{array}$$

(17) $$\begin{array}{ccc} \Theta & = & \arcsin2(e_{0}e_{2}-e_{1}e_{3}) \end{array}$$

(18) $$\begin{array}{ccc} \Psi & = & \arctan\frac{2(e_{0}e_{3}+e_{1}e_{2})}{e_{0}^{2}+e_{1}^{2}-e_{2}^{2}-e_{3}^{2}} \end{array}$$

Using the transformation matrix from (15) can be used in the second kinematic equation bounding linear velocities in $O_{n}$ coordinate system with velocities in $O_{b}$ coordinate system.

(19) $$\left[\begin{array}{c} {\dot{x}}_{n}\\ {\dot{y}}_{n}\\ {\dot{z}}_{n} \end{array}\right]=\Lambda \left[\begin{array}{c} U\\ V\\ W \end{array}\right]$$

The initial quaternion can be determined from the initial orientation angles by means of equations [29,30,31]:

(20) $$\begin{array}{ccc} e_{0} & = & \cos\frac{\Phi }{2}\cos\frac{\Theta }{2}\cos\frac{\Psi }{2}+\sin\frac{\Phi }{2}\sin\frac{\Theta }{2}\sin\frac{\Psi }{2} \end{array}$$

(21) $$\begin{array}{ccc} e_{1} & = & \sin\frac{\Phi }{2}\cos\frac{\Theta }{2}\cos\frac{\Psi }{2}-\cos\frac{\Phi }{2}\sin\frac{\Theta }{2}\sin\frac{\Psi }{2} \end{array}$$

(22) $$\begin{array}{ccc} e_{2} & = & \cos\frac{\Phi }{2}\sin\frac{\Theta }{2}\cos\frac{\Psi }{2}+\sin\frac{\Phi }{2}\cos\frac{\Theta }{2}\sin\frac{\Psi }{2} \end{array}$$

(23) $$\begin{array}{ccc} e_{3} & = & \cos\frac{\Phi }{2}\cos\frac{\Theta }{2}\sin\frac{\Psi }{2}-\sin\frac{\Phi }{2}\sin\frac{\Theta }{2}\cos\frac{\Psi }{2} \end{array}$$

#### 2.1.5. External Loads

The motion of the object is caused by the external forces and torques from aerodynamics $F_{a}$ and $M_{a}$, gravity $F_{g}$ and $M_{g}$, thrust $F_{s}$ and $M_{s}$ and reaction thrusters $F_{\mathrm{sk}}$ and $M_{\mathrm{sk}}$ [16]:

(24) $$\begin{array}{ccc} F_{b} & = & F_{a}+F_{g}+F_{s}+F_{\mathrm{sk}} \end{array}$$

(25) $$\begin{array}{ccc} M_{b} & = & M_{a}+M_{g}+M_{s}+M_{\mathrm{sk}} \end{array}$$

#### 2.1.6. Aerodynamics

Aerodynamic force and moment vectors are given with respect to point $O_{e}$, so with respect to point $O_{b}$ they are given as:

(26) $$F_{a}=\left[\begin{array}{c} X_{a}\\ Y_{a}\\ Z_{a} \end{array}\right]$$

(27) $$M_{a}=\left[\begin{array}{c} L_{a}\\ M_{a}\\ N_{a} \end{array}\right]=M_{a,O_{e}}+r_{e}\times F_{a}$$

where $r_{e}=r_{\mathrm{we}}-r_{\mathrm{wC}}+r_{C}$ is the vector describing the location of point $O_{e}$ with respect to point $O_{b}$, $r_{w}e$ is the position of point $O_{e}$ with respect to rocket’s base and $r_{\mathrm{wC}}$ is the position of point $O_{b}$ with respect to the rocket’s base. Aerodynamic force and moment are given as:

(28) $$F_{a}=\frac{1}{2}\rho {\left|V_{b}\right|}^{2}S\left[\begin{array}{c} C_{X}\left(\alpha ,\beta ,Ma\right)\\ C_{Y}\left(\alpha ,\beta ,Ma\right)\\ C_{Z}\left(\alpha ,\beta ,Ma\right) \end{array}\right]$$

(29) $$M_{a,O_{e}}=\frac{1}{2}\rho {\left|V_{b}\right|}^{2}Sd\left[\begin{array}{c} C_{l}\left(\alpha ,\beta ,Ma\right)\\ C_{m}\left(\alpha ,\beta ,Ma\right)\\ C_{n}\left(\alpha ,\beta ,Ma\right) \end{array}\right]$$

where ρ is the air density, *S* is the rocket’s cross section area and *d* is the rocket’s diameter. Aerodynamic incidence angles are given as [29,30]:

(30) $$\begin{array}{ccc} \alpha & = & \arctan\frac{W}{U} \end{array}$$

(31) $$\begin{array}{ccc} \beta & = & \arcsin\frac{V}{|V_{b}|} \end{array}$$

(32) $$\begin{array}{ccc} Ma & = & \frac{|V_{b}|}{a} \end{array}$$

where α is the angle of attack, β is the sideslip angle, and Ma is the Mach number given in (), where *a* is the local speed of sound. The aerodynamic coefficients are [9,32]:

(33) $$\begin{array}{c} C_{X}=\left(C_{X_{base\;0}}+C_{X_{base\;\alpha^{2}}}\alpha^{2}+C_{X_{base\;\beta^{2}}}\beta^{2}\right)+\left(C_{X_{eng\;0}}+C_{X_{eng\;\alpha^{2}}}\alpha^{2}+C_{X_{eng\;\beta^{2}}}\beta^{2}\right)\delta_{eng}\\ C_{Y}=C_{Y_{0}}+C_{Y_{\beta }}\beta \\ C_{Z}=C_{Z_{0}}+C_{Z_{\alpha }}\alpha \\ C_{l}=C_{l_{0}}+\left(C_{l_{p_{0}}}+C_{l_{p_{\alpha^{2}}}}\alpha^{2}+C_{l_{p_{\beta^{2}}}}\beta^{2}\right)\frac{Pd}{2|V_{b}|}\\ C_{m}=C_{m_{0}}+C_{m_{\alpha }}\alpha \\ C_{n}=C_{n_{0}}+C_{n_{\beta }}\beta \end{array}$$

where $C_{X_{0}}$ is zero-longitudinal axial force coefficient, $C_{Y_{\beta }}$ is side force with angle of sideslip derivative, $C_{Z_{\alpha }}$ is normal force with respect to angle of attack derivative, $C_{l_{0}}$ is spin driving rolling moment coefficient and $C_{l_{p}}$ is spin damping derivative. $C_{m_{\alpha }}$ is pitching moment with respect to angle of attack derivative, $C_{n_{\beta }}$ is yawing moment derivative with respect to sideslip angle. $C_{m_{q}}$ is pitching moment coefficient derivative with pitch rate and $C_{n_{r}}$ is yawing moment coefficient derivative with yaw rate. $\delta_{e}$ is the parameter that describes the main motor state ($\delta_{e}=0$ for active phase of flight and $\delta_{e}=1$ after main motor burnout, for gliding flight). When the main motor operates the projectile base drag is lower than after main motor burnout. $C_{X_{0}}$ was obtained for two system configurations (main motor on/off) and $\delta_{e}$ is used in a simulation to switch between aerodynamic data tables. Aerodynamic coefficients were obtained using commercially available software PRODAS (Projectile Rocket Ordnance Design & Analysis System). These coefficients were implemented into the Simulink model using Lookup-Table methodology. The aerodynamic coefficients are presented in Figure 2.

#### 2.1.7. Gravity

Gravitational acceleration vector in the gravitational coordinate system is given as $g={\left[\begin{array}{ccc} 0 & 0 & g_{0} \end{array}\right]}^{T}$. Gravitational acceleration is assumed constant and consistent with WGS-84 reference model [25], i.e., $g_{0}=9.80665$ m/s${}^{2}$. Gravitational force and torques are given as:

(34) $$F_{g}=T_{b}^{g}m\left[\begin{array}{c} 0\\ 0\\ g_{0} \end{array}\right]$$

(35) $$M_{g}=r_{C}\times F_{g}$$

where $T_{b}^{g}$ is the transformation matrix from gravitational to body coordinate system, given as:

(36) $$T_{g}^{b}=\left[\begin{array}{ccc} \cos\Theta \cos\Psi & \cos\Theta \sin\Psi & -\sin\Theta \\ \sin\Phi \sin\Theta \cos\Psi -\cos\Phi \sin\Psi & \sin\Phi \sin\Theta \sin\Psi +\cos\Phi \cos\Psi & \sin\Phi \cos\Theta \\ \cos\Phi \sin\Theta \cos\Psi +\sin\Phi \sin\Psi & \cos\Phi \sin\Theta \sin\Psi -\sin\Phi \cos\Psi & \cos\Phi \cos\Theta \end{array}\right]$$

#### 2.1.8. Thrust

Thrust vector, which can deviate from the rocket’s longitudinal axis by angle $\Theta_{T}$ in pitch plane and $\Psi_{T}$ in the yaw plane, is given as [16]:

(37) $$F_{s}=F_{p}\left(t\right)\left[\begin{array}{c} \cos\Theta_{T}\cos\Psi_{T}\\ \cos\Theta_{T}\sin\Psi_{T}\\ -\sin\Psi_{T} \end{array}\right]$$

where $F_{p}\left(t\right)$ is the instantaneous value of the thrust force. Torque from the thrust force with respect to $O_{b}$ is given as:

(38) $$M_{s}=\left(-r_{\mathrm{wC}}+r_{C}\right)\times F_{s}$$

#### 2.1.9. Correction Thrusters

For the gasodynamic control system comprised of a set of identical correction thrusters placed radially in a set of parallel layers, the thrust and torque from the thrusters are given as:

(39) $$F_{{\mathrm{sk}}_{i,j}}=F_{p_{sk}}\left(t\right)\left[\begin{array}{c} 0\\ \sin\Phi_{i,j}\\ -\cos\Phi_{i,j} \end{array}\right]$$

(40) $$M_{{\mathrm{sk}}_{i,j}}=\left(r_{{\mathrm{sk}}_{i,j}}-r_{\mathrm{wC}}+r_{C}\right)\times F_{{\mathrm{sk}}_{i,f}}$$

where $F_{p_{sk}}\left(t\right)$ is the instantaneous thrust force of the thruster, index i=1,⋯,M is the layer number, index j=1,⋯,N is the number of a thruster in a particular layer, $\Phi_{i,j}$ is the azimuth angle of a thruster is a particular layer, $r_{{\mathrm{sk}}_{i,j}}$ is the vector describing the position of the layer with respect to the rocket’s base, measured from the base in the direction of the rocket’s axis. The total force and torque generated by the gasodynamic control system is given as: (41) and (42)

(41) $$F_{\mathrm{sk}}=\sum_{i=1}^{M}\sum_{j=1}^{N}F_{{\mathrm{sk}}_{i,j}}$$

(42) $$M_{\mathrm{sk}}=\sum_{i=1}^{M}\sum_{j=1}^{N}M_{{\mathrm{sk}}_{i,j}}$$

**Figure 3:**
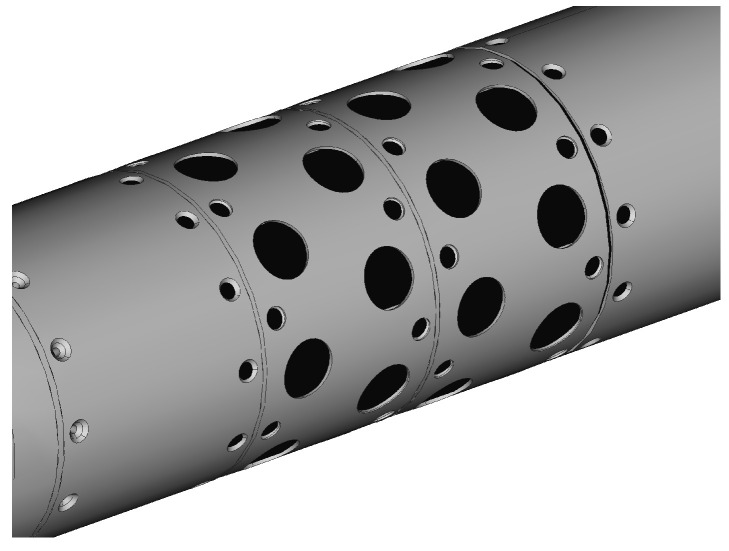
Configuration of the lateral thrusters module.

For the simulation purposes it was assumed that the projectile is equipped in a modular unit (Figure 3) composed from 32 solid propellant lateral thrusters and placed before the center of mass of the missile. These thrusters are set into a 4 arrays with 8 motors in each layer. Each of the thrusters might by used only once. The mass of the propellant in the single motor is approximately 0.005 kg so it is reasonable to assume that the ignition does not influence the mass and inertia projectile properties. The aerodynamic interference effects of the thrusters with the external flow were also omitted.

#### 2.1.10. Inertial Parameters

The instantaneous mass of the rocket is given as:

(43) $$m\left(t\right)=m_{0}-\frac{m_{p}}{I_{c}}\int_{t_{0}}^{t}F_{p}\left(t\right)dt$$

where $m_{0}$ is the starting mass of the rocket at time $t_{0}$, $m_{p}$ is the mass of the propellant and $I_{c}$ is the total impulse given as:

(44) $$I_{c}=\int_{t_{0}}^{t_{k}}F_{p}\left(t\right)dt$$

where $t_{k}$ is the time of propellant burnout. During the powered flight, the rocket’s mass center position vector $r_{\mathrm{wC}}$ measured from the rocket’s base is changing according to:

(45) $$r_{\mathrm{wC}}=\left[\begin{array}{ccc} x_{cg}\left(t\right)=x_{cg_{0}}-\frac{x_{cg_{0}}-x_{cg_{k}}}{I_{c}}\int_{t_{0}}^{t}f_{p}\left(t\right)dt & 0 & 0 \end{array}\right]$$

where $x_{cg_{0}}$ is the center of mass position on the $O_{b}x_{b}$ axis during launch and $x_{cg_{k}}$ is the center of mass position on the $O_{b}x_{b}$ axis after the propellant burnout. The change of moments of inertia can be express as:

(46) $$I_{ij}\left(t\right)=I_{ij_{0}}-\frac{I_{ij_{0}}-I_{ij_{k}}}{I_{c}}\int_{t_{0}}^{t}F_{p}\left(t\right)dt$$

where $I_{ij_{0}}$ is the moment of inertia tensor component during launch and $I_{ij_{k}}$ is the moment of inertia tensor component after the propellant burnout.

#### 2.1.11. Atmosphere Model

The air density, temperature, and the speed of sound are calculated according to the International Standard Atmosphere model [24].

(47) $$\rho =\rho_{0}{\left(1-\frac{h}{44300}\right)}^{4.256}$$

(48) $$T=T_{0}-0.0065h$$

(49) $$a=a_{0}\sqrt{\frac{T}{288}}$$

The reference values of these thermodynamic parameters are taken for the troposphere: $\rho_{0}=1.225$ kg/m${}^{3}$, $T_{0}=288.15$ K, $a_{0}=340.3$ m/s and $h=-z_{n}$ is the height in meters. It was assumed that the flight take place in the steady state atmosphere (wind speed was set to 0 m/s).

#### 2.1.12. Inertial Measurement Unit Model

It was assumed that the rocket’s is equipped with the strapdown Inertial Measurement Unit with the three-axis accelerometer and three-axis gyroscope, and these are the only sources of information about rocket position, velocity and orientation. Abovementioned design requirements are quite difficult to fullfill due to errors. Pure inertial navigation has a tendency due to drift. These drift errors might be reduced using integration with GPS receivers. Also, additional sensors like might be used to improve the system acccuracy. Magnetometers measurement are imprecise because the projectile and launcher structure are made from steel alloys. The IMU is intended for a projectile that spins about the longitudinal axis of symmetry. Photodiode sensors could be used to measure the projectile roll rate.

An example of IMU that is suitable for the considered application is Micro-Electro-Mechanical-Systems based HG1930. The mass of this device is approximately 0.16 kg and power consumption less than 3 W. The operating temperature range is from $-54{\;}^{{}^{\circ}}$C up to $+85{\;}^{{}^{\circ}}$C. The gyroscopes ranges are up to 7200 deg/s in the X axis and 1440 deg/s in the Y and Z axes (X axis range must be significanlty higher than Y and Z due to projectile axial spin). Accelerometers operating range is up to 85 g in the X axes and 35 g in the Y and Z axes. High measurement range for X axis results from acceleration caused by main motor. This device requires supply voltage of 5 V. The IMU might be connected with the central onboard computer using military standard RS-422 serial interface. The maximum rate of data transmission for control purposes is 600 Hz. This measurement device is placed in front of the missile center of mass (between main motor unit and lateral thrusters module).

Accelerometers model

The acceleration of the rocket’s center of mass in $O_{b}x_{b}y_{b}z_{b}$ coordinate system is:

(50) $$a=\left[\begin{array}{c} a_{x}\\ a_{y}\\ a_{z} \end{array}\right]=\frac{F_{b}}{m}$$

In the general case, the accelerometer position need not to coincide with the center of mass of the rocket. Therefore, the center of mass acceleration must be recalculated to the point of accelerometers’ mounting:

(51) $$a_{\mathrm{IMU}}=a+\Omega \times \left(\Omega \times r_{\mathrm{wz}}\right)+\dot{\Omega }\times r_{\mathrm{wz}}-g$$

where $r_{\mathrm{wz}}=r_{\mathrm{wC}}-r_{\mathrm{IMU}}$ is the position of the IMU with respect to center of mass and $r_{\mathrm{IMU}}$ is the IMU position with respect to the rocket’s base. As a next step, the model of the sensor’s errors was included, which is comprised of scale factors $s_{x},s_{y},s_{z}$, cross-coupling $c_{xy},c_{xz},c_{yz}$ and biases $b_{x},b_{y},b_{z}$:

(52) $${\hat{a}}_{\mathrm{IMU}}=\left[\begin{array}{ccc} s_{x} & -c_{xy} & c_{xz}\\ c_{xy} & s_{y} & -c_{yz}\\ -c_{xz} & c_{yz} & s_{z} \end{array}\right]a_{\mathrm{IMU}}+\left[\begin{array}{c} b_{x}\\ b_{y}\\ b_{z} \end{array}\right]$$

Accelerometer is treated as a second-order dynamic system:

(53) $$a_{\mathrm{meas}}=\frac{\omega_{n_{acc}}^{2}}{s^{2}+2\xi_{acc}\omega_{n_{acc}}s+\omega_{n_{acc}}^{2}}{\hat{a}}_{\mathrm{IMU}}$$

where $\xi_{acc}$ is the accelerometer damping coefficient and $\omega_{n_{acc}}$ is the accelerometer natural frequency ($\xi_{acc}=0.707$ and $\omega_{n_{acc}}=7600$). The last step was to include the sensor noise, assumed as white noise with known standard deviation and zero mean, and output saturation.

##### Gyroscopes Model

The gyroscope output does not depend on the gyroscopes’ position inside the rocket. Therefore, there is no need to transform its output to the center of mass. The gyroscope errors’ model includes scale factor, cross-coupling, bias, and sensitivity to accelerations, given by the gyroscopes’ sensitivity matrix G:

(54) $$\Omega_{\mathrm{IMU}}=\left[\begin{array}{ccc} s_{x} & -c_{xy} & c_{xz}\\ c_{xy} & s_{y} & -c_{yz}\\ -c_{xz} & c_{yz} & s_{z} \end{array}\right]\Omega +\left[\begin{array}{c} b_{x}\\ b_{y}\\ b_{z} \end{array}\right]+G\left[\begin{array}{c} a_{x}\\ a_{y}\\ a_{z} \end{array}\right]$$

Gyroscope is also treated as a second-order system:

(55) $$\Omega_{\mathrm{meas}}=\frac{\omega_{n_{gyro}}^{2}}{s^{2}+2\xi_{gyro}\omega_{n_{gyro}}s+\omega_{n_{gyro}}^{2}}\Omega_{\mathrm{IMU}}$$

where $\xi_{gyro}$ is the gyroscope damping coefficient and $\omega_{n_{gyro}}$ is the gyroscope natural frequency (it was assumed that $\xi_{gyro}=0.356$ and $\omega_{n_{gyro}}=7600$ Hz). The last step, as with accelerometers, was to include the sensor noise and output saturation. The values of $a_{\mathrm{meas}}$ and $\Omega_{meas}$ are sampled with the sensor sample frequency. From the measured angular velocity, the rocket’s orientation is calculated. To obtain the rocket’s velocity, the measured accelerations are firstly recalculated back to the center of mass position:

(56) $$a_{\mathrm{meas},\mathrm{CG}}=a_{\mathrm{meas}}+\Omega_{meas}\times \left(\Omega_{meas}\times r_{\mathrm{wz}}\right)+{\dot{\Omega }}_{meas}\times r_{\mathrm{wz}}$$

and then the velocity vector can be obtained by numerical integration:

(57) $${\dot{V}}_{b,\mathrm{meas}}=a_{\mathrm{meas},\mathrm{CG}}-\Omega_{meas}\times V_{b,\mathrm{meas}}+\Lambda {\left[\begin{array}{ccc} 0 & 0 & g_{0} \end{array}\right]}^{T}$$

where $V_{b,\mathrm{meas}}={\left[U_{meas},V_{meas},W_{meas}\right]}^{T}$. Velocity vector is expressed in body frame $O_{b}x_{b}y_{b}z_{b}$ so it must be transformed to the navigational coordinate system $O_{n}x_{n}y_{n}z_{n}$. The rocket’s position in the navigational coordinate system is calculated by numerical integration of velocity components:

(58) $$\left[\begin{array}{c} {\dot{x}}_{n,meas}\\ {\dot{y}}_{n,meas}\\ {\dot{z}}_{n,meas} \end{array}\right]=\Lambda \left[\begin{array}{c} U_{meas}\\ V_{meas}\\ W_{meas} \end{array}\right]$$

In order to solve the mentioned earlier navigation equations the initial attitude, velocity and position of the projectile must be known. It was assumed that these parameters are perfectly estimated using initial alignment procedure before the flight.

#### 2.1.13. Control Algorithms

Projectiles equipped in solid propellant lateral motors often have very low control authority. It means that the maneuverability of such objects is small. This fact makes the guidance process challenging. For the tests of landing dispersion analysis, two algorithms were created: Multi-Condition Control Algorithm (MCCA) and modified Proportional Navigation Guidance (mPNG). The guidance algorithm used in the MCCA is based on the reference trajectory tracking. This algorithm is discussed in detail in [33]. The main idea behind MCCA is to use the reference trajectory to minimize the hitting error just and the end of flight. Due to limited number of lateral motors it is difficult to track the trajectory along the full flight path. In MCCA approach the position error between reference trajectory and the actual projectile position is minimized after trajectory vertex, during the descending flight. It is assumed that the reference trajectory is calculated prior to launch, so the position of the target is known a priori, and implemented in the rocket’s control system prior to launch. The reference trajectory is calculated for unguided projectile in such a way the missile hits perfectly the target (miss distance at the end of nominal trajectory is 0 m). The guidance algorithm used in the mPNG algorithm is the classical Proportional Navigation Guidance [34] modified by the term accounting for the trajectory bending due to gravity. Both algorithms use the same thrusters’ ignition logic that is presented in [12,16]. Due to the rocket’s high roll angular velocity during the flight, the correction thrusters must be ignited in the right moment, which means when the rocket achieves a certain roll angle. At any moment, only one correction thruster can be ignited. The set of conditions of thrusters’ ignition, common for both algorithms are:

- Correction thruster was not used already (solid motor thrusters are single-use motors)
- The time between the last thruster ignition $t_{last}$ is greater than some limit value τ∈(0;∞)
  (59) $$t-t_{last}>\tau$$
- The correction thruster must be ignited so that the resultant thrust force was in the direction of the desired lateral displacement [35,36], which means that the absolute value of the difference between the error phase γ and thruster azimuth angle $\Phi_{i,j}$ diminished by the control prediction times $\tau_{d}$ and $\tau_{sk}$ multiplied by the roll angular velocity was lower than some limit value $\gamma_{t}$.
  (60) $$\left|\gamma -\Phi_{i,j}-\pi -P\left(\tau_{d}+\tau_{sk}\right)\right|\leq \gamma_{t}$$
- The rocket’s pitch angle must be lower or equal the threshold value $\Theta_{g}$ and the time of flight must be at least equal to the threshold value $t_{g}$
  (61) $$\Theta \leq \Theta_{g}\wedge t\geq t_{g}$$

Additional conditions for the MCCA algorithm:

- The distance between the rocket’s center of mass and the reference trajectory Γ, measured perpendicular, is greater than some limit value $\Gamma_{t}$
  (62) $$\Gamma >\Gamma_{t}$$

Additional conditions for the mPNG algorithm:

- the norm of the commanded value of the lateral acceleration $a_{\mathrm{cmd}}$ must be greater than the threshold value $a_{\mathrm{cmd},g}$
  (63) $$a_{\mathrm{cmd}}\geq a_{\mathrm{cmd},g}$$

The parameters of the control laws were determined using the expert method and parametric study: τ=0.2 s, $\tau_{d}=0.001$ s, $\tau_{sk}=0.015$ s, $\gamma_{t}=2.5$ deg, $\Theta_{g}=-10$ deg (the guidance process starts after trajectory vertex), $t_{g}=15$ s, $\Gamma_{t}=1$ m, $a_{cmd,g}=3$ m/s${}^{2}$.

### 2.2. Simulation Model

**Figure 4:**
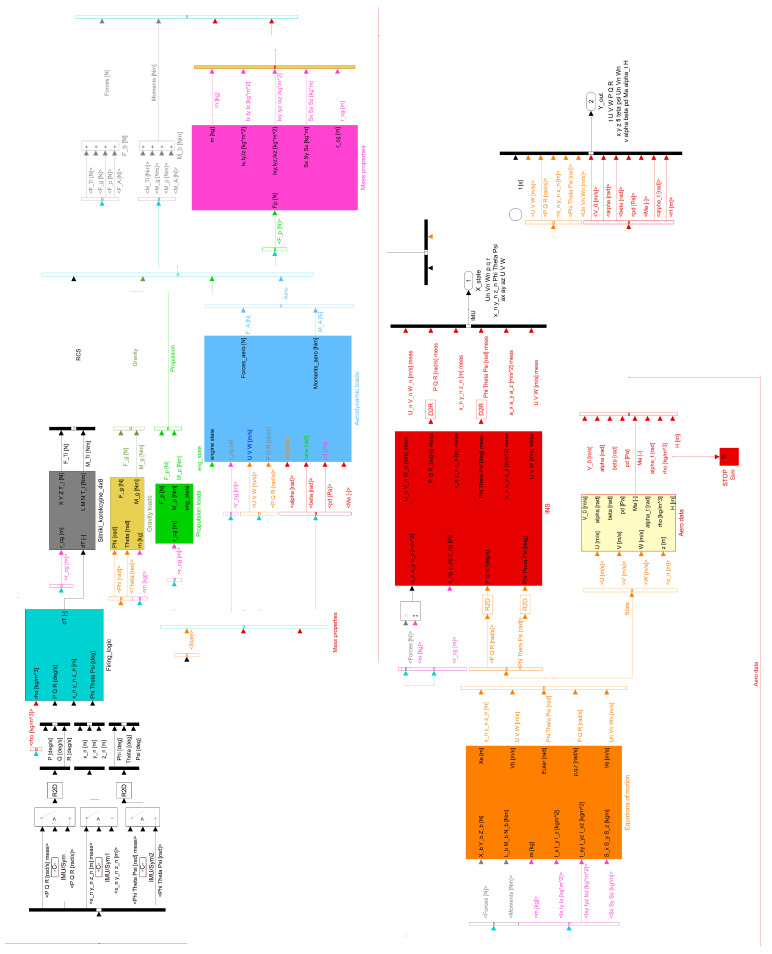
Top level architecture of the Simulink simulational model.

The mathematical model described in Section 2.1 was implemented in MATLAB/ Simulink 2020b environment. The main Simulink block model of the system is presented in Figure 4.

The program simulates the flight of the gasodynamically controlled rocket, calculates the loads from gravity, aerodynamics, thrust, and correction thrusters. It solves the set of ordinary differential equations for the rigid body with 6 degrees of freedom and variable mass. It includes the models of International Standard Atmosphere and Inertial Measurement Unit as well as the inertial navigation equation for determining the rocket’s position, orientation, and velocity. The equations of motion of the projectile were integrated using fixed step, third order Bogacki-Shampine method. The step size was set to 0.0001 s. Simulations were realized using Simulink build in option “Accelerator mode”. Marsenne-Twister algorithm [37] was used to generate in a pseudorandom way the disturbances for the Monte-Carlo simulation. The model was optimized to make the run time as short as possible. The simulation might be realized in a batch mode from the external MATLAB script.

## 3. Results and Discussion

### 3.1. Input Data for the Simulation Study

A generic rocket model was used for the simulations, which general data are provided in Table 1.

**Figure 5:**
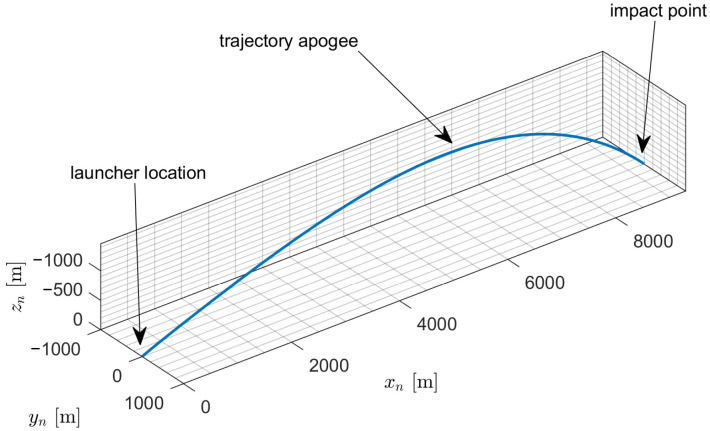
Projectile trajectory.

The missile is stabilized with four trapezoidal fins. The maximum flight velocity of this projectile is 605 m/s and maximum roll rate 4700 deg/s (these values are obtained in 3 s of flight). The missile was fired at elevation angle 25 deg. Initial velocity was set to 42 m/s and initial roll rate 1073 deg/s. Projectile was fired from the initial position (0, 0, 0) m. The reference trajectory of the projectile (unguided flight) is presented in Figure 5.

### 3.2. Initial Verification of Control Algorithms

As a first step, five deterministic cases were evaluated to test if the prepared control algorithms work as intended: no control, mPNG algorithm with and without IMU model, and MCCA algorithm with and without IMU model. To intentionally introduce aiming error it was assumed that the launch tube is not perfectly aligned with the demanded shoot direction. The initial heading error angle equaled 2 degrees. The position coordinates of the stationary target were set to (9296.54,−7.29,0) m. Table 2 presents the results of the performed cases. The first column describes the used algorithm, with the information whether the IMU model was on. The next columns present the error between the *x* and *y* components of the rocket’s and reference trajectories at the impact point, and the distance between the rocket’s landing point and the target position given as $\Delta R=\sqrt{\Delta X^{2}+\Delta Y^{2}}$.

The results shown in Table 2 indicate that the largest error was obtained for the uncontrolled flight. The projectile landed 324.5 m from the desired point and it is obvious that the target was not achieved (typical radius of destruction for rocket artillery projectiles is order of 20–40 m). Both algorithms work properly and that the mPNG control algorithm lowers the miss distance by about 92% and MCCA algorithm by about 75% on average. The IMU model errors slightly increase the miss distance for both algorithms.

### 3.3. Monte-Carlo Simulations

Next, to test the influence of various uncertainties on the performance of control algorithms and the resulting landing dispersion, a few sets of simulations were performed. Tested were uncertainties in aerodynamic data, thrust parameters, and initial conditions. For every uncertainty, again five cases were simulated: no control, mPNG algorithm with and without IMU model, and MCCA algorithm with and without IMU model. Every case took 1000 runs, using the Monte Carlo method, giving a total of 15 thousand runs. As a merit of accuracy, the Circular Error Probable CEP was used. It gives information about the radius of a circle inside which 50% of landing points are located.

#### 3.3.1. Aerodynamic Parameters Uncertainties

**Figure 6:**
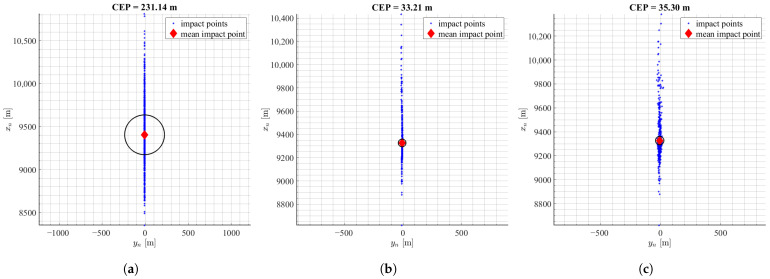

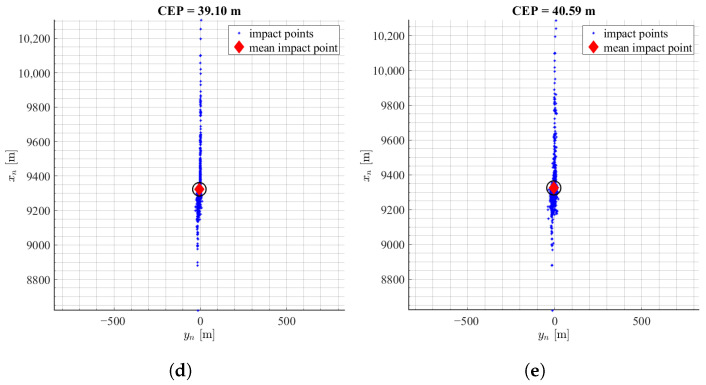
Results of landing point dispersion with uncertainties of aerodynamic data: (**a**) no control, (**b**) mPNG, (**c**) mPNG + IMU, (**d**) MCCA, (**e**) MCCA + IMU.

The first set of Monte Carlo simulations consisted of uncertainties in aerodynamic data. It was assumed that the normal distribution standard deviation of all aerodynamic parameters was equal to σ=0.2. Next, the maps of impact points were obtained. The Figure 6 presents the results of the performed simulations. On the horizontal axis there is crossrange and on the vertical axis range of the projectile.

In the not controlled case, the CEP was equal to 231.14 m. Ideal case of mPNG and MCCA algorithms (IMU model off) achieved 85.6% and 83.1% miss distance reduction respectively and with the IMU model on 84.5% for mPNG and 82.4% for MCCA. Much bigger dispersion is observed along the rocket’s flight path, because the drag coefficient uncertainties have the biggest influence on the range. Small directional dispersion is mostly caused by the IMU model errors. The achieved miss distance reduction for both algorithms were very similar.

#### 3.3.2. Thrust Curve Uncertainties

**Figure 7:**
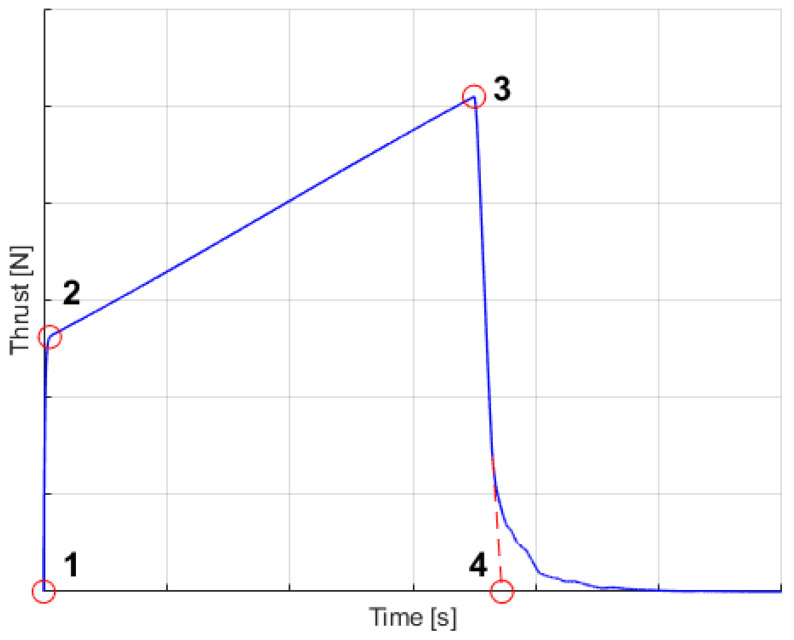
Thrust curve.

The next set of simulations consisted of uncertainties in the thrust data. For simplicity, it was assumed that the thrust curve can be approximated by the quadrilateral comprised of four characteristic pairs of points, time-thrust, presented in Figure 7. Every point was randomly chosen, using a uniform distribution, between the maximum and minimum allowable values, with additional constraints that the 4th time had to be larger that the 3rd time and that the total impulse of the thrust should lie in between allowable values. The values of the uncertainties are presented in Table 3. In this way a set of pseudorandom thrust curves was obtained as input data for the Monte-Carlo simulations (in each simulation run a different thrust curve was used).

**Figure 8:**
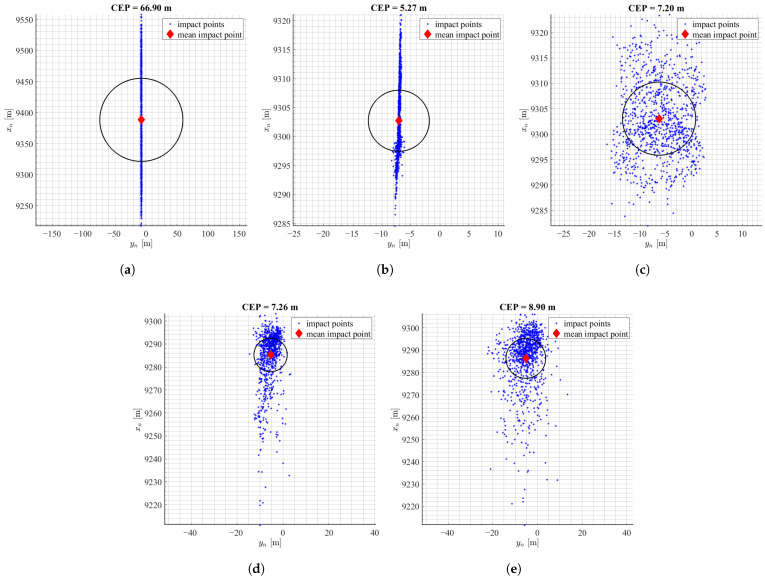
Results of landing point dispersion with uncertainties of thrust data: (**a**) no control, (**b**) mPNG, (**c**) mPNG + IMU, (**d**) MCCA, (**e**) MCCA + IMU.

The Figure 8 presents the results of the performed simulations. Again, the largest dispersion of the impact points was observed for the uncontrolled projectile. The uncontrolled flight case resulted in CEP of 66.90 m. It means, that thrust uncertainties produce smaller dispersion than uncertainties in aerodynamic parameters. This dispersion is reduced significantly in controlled shoots. Results for mPNG and MCCA algorithms without IMU achieved 92.1% and 89.2% miss distance reduction respectively and with the IMU model on 89.2% for mPNG and 86.7% for MCCA. Again, a bigger dispersion is observed in the longitudinal direction, which thrust uncertainties affect the most. Directional dispersion is a bit lower for the mPNG algorithm. Again, the results for both algorithms lied very close.

#### 3.3.3. Initial Condition Uncertainties

**Figure 9:**
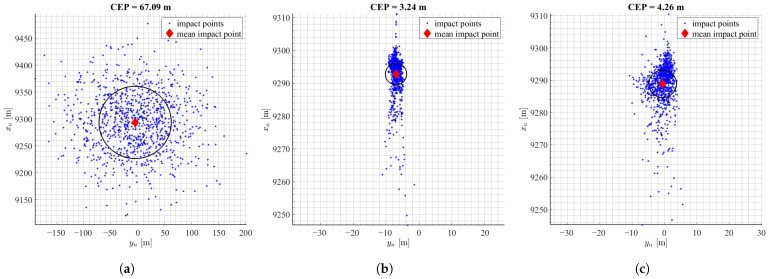

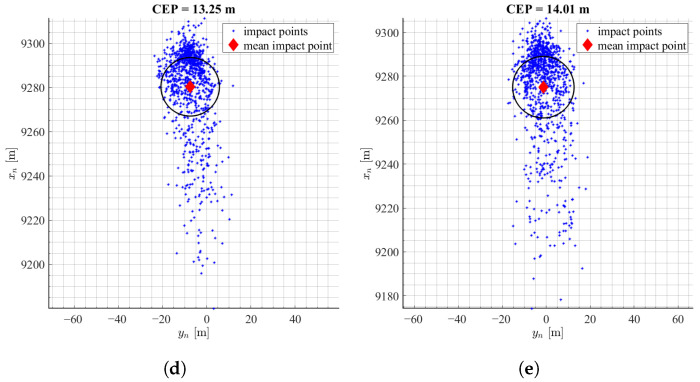
Results of landing point dispersion with uncertainties of initial conditions: (**a**) no control, (**b**) mPNG, (**c**) mPNG + IMU, (**d**) MCCA, (**e**) MCCA + IMU.

The last set of simulations consisted of uncertainties in the initial conditions. The values of the initial linear velocity vector, angular velocity vector, and orientation angles were chosen randomly, using a normal distribution, using set values of standard deviation. The Figure 9 presents the results of the performed simulations. The uncontrolled case resulted in CEP of 67.09 m and the longitudinal and directional dispersion were the same. This is a typical dispersion pattern that is obtained for rocket artillery projectiles at medium elevation angles. Results for mPNG and MCCA algorithms without IMU achieved 95.2% and 80.3% miss distance reduction respectively and with the IMU model on 93.7% for mPNG and 79.1% for MCCA. In this scenario, the miss distance reduction is in favour of the mPNG algorithm with around 10% difference in results. Directional dispersion is more affected by control than the longitudinal, which may arise from the difference in longitudinal and lateral velocity of the rocket.

Several new aspects brought by the paper might be mentioned. First, the influence of measurement errors on the resulting projectile miss distance was investigated. Second, two different guidance methods intended for lateral thrusters controlled missiles were compared for idealized and realistic case. From the obtained results it might be concluded that it is possible to achieve CEP order of several meters but to realize this goal the missile must be equipped in high-accuracy IMU.

## 4. Conclusions

Precision guided munition become more and more important in modern military conflicts. To achieve a high direct hit probability the influence of various factors on the resulting dispersion must be understood in detail. In the article, the impact point dispersion, caused by the uncertainties in aerodynamic parameters, thrust curve, initial conditions, and on-board measuring devices, for two types of control algorithms, was presented. Monte Carlo approach was used in the simulations and as the merit of dispersion the CEP was utilized.

The results showed that the use of control algorithms greatly reduces the miss distance by more than 80% in most simulated cases. From the simulations it might be concluded that there is possible to achieve CEP smaller than 8 m. For modern guided munition this is quite a realistic result (for example, 160 mm ACCULAR projectile has declared CEP < 10 m). It means that equipping the projectile with control module composed from lateral thrusters allows effectively reduce the impact points dispersion. The uncontrolled projectile might land even 200 m from the intended location in the worst-case scenario. This issue is very important in modern military applications due to the requirement of minimizing the collateral damage.

The IMU model causes a slight increase in the dispersion of about 3% in every case. The mPNG algorithm proved to be better for all simulated cases, the greatest difference between the two algorithms was observed in the initial condition case dispersion.

The developed numerical simulation might be used in the design of new measurement systems intended for missile navigation. Parametric model allows on rapid implementation of data for other missiles and IMU-s and investigate the dispersion as a function of measurement uncertainties. In this way the overall time and cost of the projectile design might be reduced.

Further works might concentrate on flight tests of the real ground-to-ground projectile and validating the model. Also wind tunnel measurements of the missile could be evaluated to obtain the aerodynamic data for a wide range of flight conditions. The influence of wind on the projectile dispersion might be also explored in detail. Hardware-in-the-loop simulation might be also considered to investigate the influence of sensor errors on the projectile hitting accuracy.

## Figures and Tables

**Figure 2:**
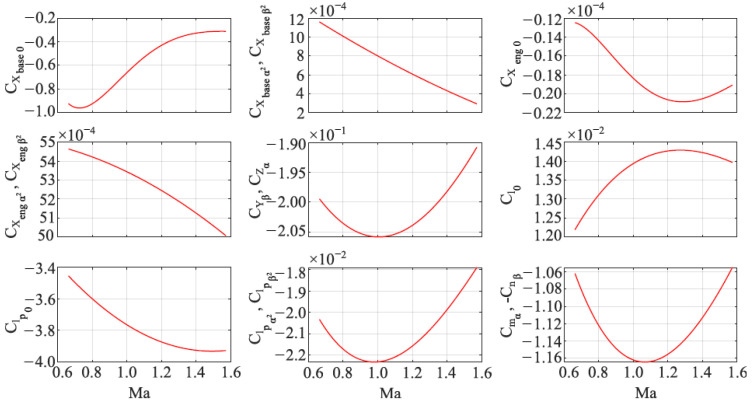
Aerodynamic coefficients.

**Table 1:** Generic rocket’s parameters.

Parameter	Value	Unit
diameter	122	mm
length	1.58	m
initial mass	22.14	kg
propellant mass	5.83	kg
initial moment of inertia $I_{xx}$	0.0422	kg·m${}^{2}$
final moment of inertia $I_{xx}$	0.0326	kg·m${}^{2}$
initial moment of inertia $I_{yy}$	11.223	kg·m${}^{2}$
final moment of inertia $I_{yy}$	9.513	kg·m${}^{2}$
initial moment of inertia $I_{zz}$	11.223	kg·m${}^{2}$
final moment of inertia $I_{zz}$	9.513	kg·m${}^{2}$
maximum thrust	7277.5	N
average thrust	3383.2	N
burn time	3.31	s
total impulse	13,529	N
correction thruster’s thrust	200	N
correction thruster’s burn time	0.03	s
number of correction thrusters per layer	8	-
number of correction thrusters’ layers	4	-

**Table 2:** The errors in landing point components.

Algorithm	ΔX[m]	ΔY[m]	ΔR[m]
None	−5.4	324.4	324.5
mPNG	−12.3	19.7	23.2
mPNG + IMU	−15.3	20.1	25.3
MCCA	−32.1	69.8	76.8
MCCA + IMU	−32.0	75.2	81.8

**Table 3:** Thrust curve uncertainties.

Point	Value	Time *t* [s]	Thrust *T* [N]
1	min	0	0
1	max	0	0
2	min	0	3600
2	max	0.15	4400
3	min	1.9	6900
3	max	2.5	8700
4	min	2.1	0
4	max	2.7	0

## Data Availability

Not applicable.
